# The Possible Role of Gut Microbiota and Microbial Translocation Profiling During Chemo-Free Treatment of Lymphoid Malignancies

**DOI:** 10.3390/ijms20071748

**Published:** 2019-04-09

**Authors:** Valentina Zuccaro, Andrea Lombardi, Erika Asperges, Paolo Sacchi, Piero Marone, Alessandra Gazzola, Luca Arcaini, Raffaele Bruno

**Affiliations:** 1 Infectious Diseases Unit, Fondazione IRCCS “San Matteo”, 27100 Pavia, Italy; zuccaro.v@gmail.com (V.Z.); andrea.lombardi02@universitadipavia.it (A.L.); erika.asperges01@universitadipavia.it (E.A.); paolo.sacchi1962@gmail.com (P.S.); alegazzola@hotmail.it (A.G.); 2 U.O.C. Microbiologia e Virologia, Fondazione IRCCS Policlinico San Matteo, 27100 Pavia, Italy; pmarone@smatteo.pv.it; 3 Department of Molecular Medicine, University of Pavia, 27100 Pavia, Italy; luca.arcaini@unipv.it; 4 Department of Hematology Oncology, Fondazione IRCCS Policlinico San Matteo, 27100 Pavia, Italy; 5 Department of Medical, Surgical, Diagnostic and Paediatric Science, University of Pavia, 27100 Pavia, Italy

**Keywords:** gut microbiota, chemo free treatment, lymphoid malignancies

## Abstract

The crosstalk between gut microbiota (GM) and the immune system is intense and complex. When dysbiosis occurs, the resulting pro-inflammatory environment can lead to bacterial translocation, systemic immune activation, tissue damage, and cancerogenesis. GM composition seems to impact both the therapeutic activity and the side effects of anticancer treatment; in particular, robust evidence has shown that the GM modulates the response to immunotherapy in patients affected by metastatic melanoma. Despite accumulating knowledge supporting the role of GM composition in lymphomagenesis, unexplored areas still remain. No studies have been designed to investigate GM alteration in patients diagnosed with lymphoproliferative disorders and treated with chemo-free therapies, and the potential association between GM, therapy outcome, and immune-related adverse events has never been analyzed. Additional studies should be considered to create opportunities for a more tailored approach in this set of patients. In this review, we describe the possible role of the GM during chemo-free treatment of lymphoid malignancies.

## 1. Introduction

The use of small molecules and immune-targeted therapies has had significant impact on the prognosis of some cancers. Data have shown that gut microbiota composition may play a significant role in determining the efficacy and safety of such therapies. So far, the relationship between microbiota and hematologic malignancies is not well understood.

The aim of this review was to describe the possible role of the gut microbiota (GM) and microbial translocation profiling during chemo-free treatment of lymphoid malignancies. A web-based search of MEDLINE (PubMed) was performed from 2009 until March 2018 in order to identify pertinent articles. We structured our term search using the following keywords: “gut microbiota; chemo free treatment; lymphoid malignancies”. This review first describes the characteristic of the gut microbiota and its relationship with both the immune system and inflammation and microbial translocation. Then, we report what is known about the interplay between microbiota cancer, hematologic disorders, and lymphoid malignancies.

## 2. Human Gut Microbiota

The human microbiota is composed of numerous micro-organisms including eukaryotes, archaea, bacteria, and viruses, which colonize the whole human body: skin surface, airways, and genital and gastrointestinal systems [1]. It consists of 10–100 trillion cells and the number of genes greatly exceeds those in the human genome [2]. Considerable effort has been invested in characterizing the human microbiota using next generation sequencing (NGS) technology: the Human Microbiome Project (HMP), launched in 2008, was designed to estimate the complexity of the microbial community at each body site to understand its potential role in human health [3].

The majority of microbes harbored in the gut (GM) are bacteria, at around 10^13^–10^14^ bacterial cells [1]. In healthy intestines, the GM is dominated by Gram-negative *Bacteroidetes* phyla and Gram-positive *Firmicutes* phyla, with small proportions of *Actinobacteria*, *Proteobacteria*, and *Verrucomicrobia* [1,4]. The GM is a dynamic ecosystem linked to age, geographical location, human lifestyle (diet), and environmental factors [5]. For this reason, identifying a stable composition of healthy GM is difficult. 

Resilience measures the extent to which, and how permanently, any kind of stress may perturb GM composition [1,6]. To better assess the community resilience, reproducible patterns of GM, called enterotypes, were identified using shotgun sequencing of fecal metagenomes from healthy individuals of European and American descent [7,8,9,10]. Three robust clusters were recognized: Enterotype 1 is rich in *Bacteroides* and *Parabacteroides* and is able to derive energy from carbohydrates and proteins (Western diet); Enterotype 2 is rich in *Prevotella* and *Desulfovibrio* and their hydrolases are specialized in the degradation of plant fibers; and Enterotype 3, the most frequent, is rich in *Ruminococcus* as well as co-occurring *Akkermansia* [5,8]. 

GM can influence physiological human homeostasis: it has metabolic functions, provides protection against pathogens, and modulates the immune response. GM modifications associated with disease are being increasingly frequently studied. Dysbiosis includes any condition that disrupts the stable composition of that GM. It can be caused by infection and by environmental factors such as antibiotics consumption or dietary changes [11,12]. Several human and animal studies showed a link between dysbiosis and disease, such as cancer, immune-related disorders, metabolic diseases, inflammatory bowel disease, pulmonary conditions, oral diseases, as well as skin and neurological disorders [4,12]. 

## 3. The Role of Gut Microbiota on the Immune System

The GM has multiple functions and the relationship with the host is regulated by a complex network of interactions. The GM is involved in energy harvest and storage and plays a role in generating nutrients from substrates indigestible by the host, such as starch and soluble dietary fiber. These products act as energy substrates for the host and, unfortunately, as effectors of immune responses and tumorigenesis [13,14].

The crosstalk between GM and the immune system is intense and complex. The gastrointestinal (GI) tract is one of the body niches where the external environment meets the internal one. The GI tract is composed of enterocytes covered by mucous, immunoglobulins A, and glycocalyx, which separates the luminal environment from the lymphoid tissue. To reach the lymphoid tissue, antigens pass through the cells (transcellular movement mediated by pumps and channels) or through paracellular compartments (tight junctions). The intestinal barrier then acts as a physical barrier and its integrity is crucial for maintaining the balance between health and disease [15]. The intestinal barrier consists of another structure: the immunologic barrier, which is composed of lymphoid cells and humoral factors such as dendritic cells, macrophages, granulocytes, mast cells, B and T cells, and CD4+CD25+ cells. The GM is a part of a third intestinal barrier, called the biological barrier, and it includes several antimicrobial molecules acting as a defense against pathogens [15]. For these reasons, both the GM and the intestinal barrier are defined as the “missing organs” of the human body [16].

Even in healthy intestine, commensal bacteria influence immune homeostasis. Pattern recognition receptors, like toll-like receptors (TLRs), present on the enterocytes recognize pathogen associated molecular patterns (PAMPs) of commensal bacteria, promoting the initiation of the inflammatory response [1,17] by the release of nuclear factor kappa-light-chain-enhancer of activated B cells (NF-κB), which activates a variety of genes coding for chemokines, cytokines, acute phase proteins, and other effectors of the humoral immune response [18]. Some bacteria can produce metabolites, such as short-chain fatty acids (SCFAs) and reactive oxygen species (ROS), able to activate T cells, and regulatory T cells (Tregs) versus Th17 phenotype. When dysbiosis occurs, the resultant pro-inflammatory environment can aggravate the inflammatory status, triggering the recruitment of immune effector cells and the shedding of additional pro-inflammatory cytokines [1,13]. Beyond the recruitment of immune cells, the GM shapes global immune cell repertoires by modulating the differentiation of T cell populations into different types of helper cells (Th): Th1, Th2, and Th17, or into Tregs [19,20]. SCFAs are suppressors of nuclear NF-κB, interleukein-6 (IL-6), and tumor necrosis factor α (TNF- α) and enhance the production of IL-10. Through this mechanism, SCFAs promote the generation of Th1, Th17, and IL-10+ cells and decrease the proliferation of T and B cells, whereas a specific type of SCFAs, butyrate, enhances T-cell apoptosis [19]. Next to the proinflammatory role, the GM may have also a protective role. Tregs limits the aberrant inflammatory response and several studies have reported how the microbial community promotes the differentiation of anti-inflammatory regulatory T cells: Mazmanian et al. [20] demonstrated the role of *Bacteroides fragilis* in suppressing the production of IL-17 and in protecting against potential inflammatory reactions initiated by bacterial antigens.

Several studies and animal models have demonstrated that GM composition leads to a proper maturation of the immune system and the production of haemopoietic cells [19]. *Clostridiales* species, for example, suppress immune response by promoting Tregs polarization and IL-10 production [21,22]. *Enterococcus hirae* increase the level of Th17 and then stimulate the immune response [21,23]. 

While there is accumulating evidence on the role of GM in gut local immunity, more data are needed to confirm the relationship between the GM and systemic immunity and inflammation. For example, Ichinohe et al. [24,25] showed how the consumption of broad-spectrum antibiotics seems to reduce the T and B cell response against intranasal infection due to the influenza virus by promoting the inflammasome-mediated induction of IL-1β and IL-18 secretion. Commensal-derived peptidoglycan seemed to improve the killing of *Streptococcus pneumoniae* and *Staphylococcus aureus* by bone-marrow derived neutrophils in a **nucleotide-binding oligomerization domain-containing protein 1** (NOD1), an intracellular pattern-recognition receptor. [24,26]. 

## 4. The Relationship Between GM and Microbial Translocation

Microbial translocation (MT) is defined as the non-physiological passage of the GI bacteria from the gut lumen to the local mesenteric lymph nodes [27]. In physiological conditions, bacteria are phagocytized before they reach the lymph nodes. If the intestinal barrier functioning is reduced with increased intestinal permeability as a result of the impairment of local immunity, such as in the case of dysbiosis, MT can occurs [28].

MT has been extensively studied in animal models and the endpoint used to quantify MT is the number of organisms cultured in the regional lymph nodes [28]. In recent years, the association between GM, MT, and immune activation was reported by several studies in humans, particularly in HIV-infected patients [27,29]. Individuals with multiple sclerosis (MS) are characterized by low-grade translocation of bacteria from the intestines into the systemic circulation. Some authors speculated on the possibility that MT contributes to the development of the disease [30]. Facultative intracellular pathogens are able to resist phagocytic killing and are mainly responsible for MT. Several MT surrogate markers are described in literature; the most relevant is lipopolysaccharide (LPS). Soluble CD14 (sCD14) is also widely used; it is a biomarker of monocyte activation not specific for MT [27]. Other surrogate MT markers are bacterial DNA fragments and LPS binding protein (LBP), which binds to LPS and presents it to CD14 and TLR-4. 

The relationship between GM and MT is important because translocating bacteria and microbial components may aggravate a pre-existing inflammatory status. To the best of our knowledge, there are no studies on the links between modifications of GM composition, MT, and inflammatory status. 

## 5. Gut Microbiota and Cancer

Considering the above, the interest in the potential crosslink between dysbiosis and disease, in particular with cancer, is not surprising. 

Several epidemiological studies demonstrated the association between intra-abdominal infections, the use of antibiotics, the consequent dysbiosis, and an increased incidence of colorectal cancer. Carcinogen-induced models of tumorigenesis highlighted the oncogenic effects of some bacterial metabolites and ROS species [11]. Oncogenic effects of dysbiosis act locally and systematically; however, it is complicated to prove these relationships. The GM not only have an effect on carcinogenesis, but also on the pharmacology and the side effects of anticancer therapies [11]. 

We now focus on the potential role of the GM in the response to chemo-free treatments. Chemo-free cancer therapy is a novel therapeutic approach that aims to control the malignancy by taking advantage of the immune system’s physiological activity. Chemo-free agents include cytokines, checkpoint inhibitors, agonists of co-stimulatory receptors, T cells manipulators, oncolytic viruses, vaccines, and therapies directed at other cell types [31]. Currently, the most widely studied and used checkpoint inhibitors are: monoclonal antibodies (mAbs) that target programmed cell death protein 1 (PD-1) (pembrolizumab, nivolumab), its ligand PD-L1 (atezolizumab, avelumab, durvalumab), and the cytotoxic T-lymphocyte-associated protein 4 (CTLA-4) (ipilimumab, tremelimumab). These molecules are approved and indicated for different cancers, such as advanced melanoma, non-small-cell lung cancer (NSCLC), and renal cell carcinoma [31,32]. They have dramatically improved patients’ survival; however, the beneficial effects were ascertained only in a subgroup of patients [33]. In a mice model, Sivan et al. [33,34] supplied robust evidence about the impact of the GM on the efficacy of PD-L1 blockage: Bifidobacterium-treated mice improved tumor control in contrast with non-Bifidobacterium-treated mice, suggesting the possibility of enhancing the anti-tumor efficacy of anti-PD-L1 with probiotics. Matson et al. [33,35] confirmed these data in human studies: they found that in patients with metastatic melanoma, ***Bifidobacterium*** *longum*, *Collinsella aerofaciens*, and *Enterococcus faecium* were more abundant in the stool samples (collected before the start of therapy) of chemo-free therapy responders, thus supporting the anti-tumor effects of the Bifidobacterium species. In another set of metastatic melanoma patients receiving anti-PD1 therapy, Wargo et al. [33,36] found that responders had a high bacterial diversity and an abundance of *Ruminococcaceae,* whereas non-responders showed a higher percentage of Bacteroidales.

Many other studies were designed with different patients to better characterize GM composition and its contribution in chemo-free therapies efficacy and toxicity [17]. The research data have increased on the potential role of GM modulation through diet, administration of probiotics, and fecal microbial translocation to improve response to therapy. 

In their review, Gopalakrishnan et al. [17] summarized the ongoing and planned clinical trials designed to evaluate the possible GM manipulation to enhance responses to cancer immunotherapy. Several approaches were presented involving fecal transplantation, administration of probiotics, physical activities, and specific integration within a normal diet. Two trials investigating the role of GM in colorectal cancer have concluded and showed how patients with colon cancer harbor a distinct microbiota signature and how probiotics administration can modify the cytokines expression profile.

## 6. Gut Microbiota and Hematologic Disorders

**Figure 1:**
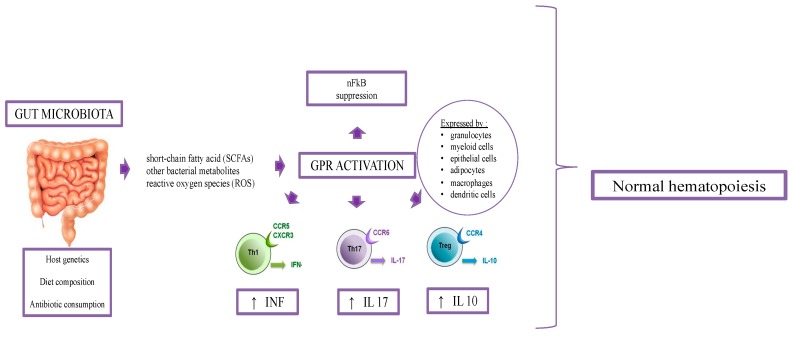
Gut microbiome composition in hematopoiesis. SCFAs activates several G-protein-coupled cell surface receptors, such as GPR43, GPR109a and C4, expressed by granulocytes, some myeloid cells epithelial cells, adipocytes, macrophages, and dendritic cells. SCFAs are responsible for promoting the generation of Th1, Th17, and IL-10+ cells and for decreasing the proliferation of T and B cells.

GM seems to have an impact on the products of the hematopoietic systems. Although the underlying mechanisms are unclear, the pool of the bone marrow myeloid cells is strongly correlated with GM composition [19]. Balmer et al. [37] compared the number of myeloid cells, mature monocytes, and granulocyte-monocyte progenitor between germ-free mice and specific pathogen-free (SPF) mice. SCFAs also play a fundamental role in the hematological setting: the product activates several G-protein-coupled cell surface receptors, such as GPR43, GPR109a, and C4, expressed by granulocytes, some myeloid cells epithelial cells, adipocytes, macrophages, and dendritic cells. As discussed above, SCFAs promote the generation of Th1, Th17, and IL-10+ cells and decrease the proliferation of T and B cells (Figure 1) [19].

Considering the close interplay between the immune system and the GM, the lymphoid tissue is involved in the oncogenic process. One example of this is mucosal-associated lymphoid tissue (MALT) lymphoma, which is strongly associated with the presence of certain bacteria, such as *Helicobacter pylori* [38] (Table 1).

As discussed above, dysbiosis can stimulate local gut immunity and generate a pro-inflammatory environment. Among the immune effector cells that participate in local immunity, lymphocytes play a key role in responding to microbial perturbation. Bacterial metabolites, such as SCFAs, seem to influence cell type recruitment and hematopoiesis [43,44]. Given the role of GM in normal hematopoiesis, alterations in the GM composition are associated with hematologic disorders. Some GM compositions are associated with the promotion or neutralization of mutagens and oxidative stress [38]. Some enterotypes or dysbiosis can act as antigens and as chronic stimulation to immune cells, leading the potential expansion of B cells [38]. A correlation between microbial tanslocation and lymphoma and blood cancer exists: *Borrelia burgdorferi* was associated with cutaneous B-cell non-Hodgkin lymphoma [43,45], and *Chlamydophila psittaci* was been detected in various non-gastrointestinal organs in patients with MALT [43,46].

Despite the strong rational linking gut immunity perturbation to lymphomagenesis, there is a paucity of animal models supporting this hypothesis. However, one example was provided by the inoculation of segmented filamentous bacteria in the intestine of mice, which leads to a change in T cell activity eliciting a range of responses including increases in IL-10, IL-17, and IFN-γ [47]. Another animal model showed an alternative underlying mechanism of lymphomagenesis due to GM composition: mice with a restricted microbiota showed an increase in CD8+ T cells and, consequently, a decrease in B cells in the marginal zone through a cytolytic mechanism. [38,48]. In a mouse model, Scheeren et al. [49,50] showed that high-level expression of IL-21 is critically associated with the expansion of mature B cells, leading to potential development of Hodgkin disease, multiple myeloma, chronic lymphocytic leukemia, Waldenstrom macroglobulinemia, and angioimmunoblastic T-cell lymphoma. Rajagopala et al. [51] compared GM composition in pediatric and adolescent patients with acute leukemia with that of their sibling controls. The GM profiles of both groups were dominated by *Bacteroides*, *Prevotella*, and *Faecalibacterium*, but the diversity of the patient group was significantly lower than that of the control group.

Once it was ascertained that the GM composition impacts normal hematopoiesis, researchers’ efforts were directed toward understanding the role of the microbiota in mediating treatment response and the outcomes in response to therapy. Concerning lymphoid malignancies, chemo-free agents represent the newest approach in the treatment of lymphoproliferative disorders [52]. However, some patients are still not responding to chemo-free therapy, the efficacy of which could be limited by the occurrence of immune related adverse events (irAEs). Since most of the side effects of these new molecules are gut-related (i.e., colitis and diarrhea), the GM could be implicated in the genesis and development of such adverse reactions. However, to the best of our knowledge, no studies have ever explored GM alteration in patients with lymphoproliferative disorders treated with chemo-free therapies and its association with the outcome and immune-related adverse events. In a recent multicentric study conducted by Peled et al. [53], patients undergoing hematopoietic cell transplantation (HCT) at four institutions in three continents presented a similar GM composition, whereas the GM differed when compared with those of healthy people. Pre-HCT microbiota injury seemed to predict poor overall survival. 

As discussed above, beyond bacterial species, almost 1500 virotypes colonize the gut lumen (10^9^ virus-like particles (VLPs) per gram of human feces) [54]. This aspect is interesting when we consider the association of lymphomas with specific microorganisms. The viral infection–lymphoma relationship was described between HCV and B-cell clonal expansion, EBV and endemic Burkitt lymphoma and post-transplantation lymphoproliferative disorder (PTLD), and human herpesvirus 8 (HHV-8) and multicentric Castleman disease [19]. For this reason, more efforts should be devoted to elucidate the potential link between gut virome and disease, maybe with the creation of a human virome project [54].

## 7. Potential Correlation and Clinical Implication

Given what was described above, we hypothesize that the immunostimulatory and antitumor effects of BCRi in patients with lymphoid malignancies could be influenced by distinct gut microbiota compositions. Therefore, the study of the gut microbiota in these patients might be important for recognizing different enterotypes able to distinguish among patients who have and have not achieved a clinical response and those at greater risk to experience immune-related adverse events (irAEs).

## 8. Conclusions and Future Directions

Thanks to the progress in the fields of biotechnology, genetics, and genomics, clinicians can use the analysis of big data as an additional tool to choose the best decision-making algorithm; this is called precision medicine [16,55]. 

Specific gut microbiota states are reportedly associated with autoimmune disorders and, although the relationship between microbes and host immune responses suggests an association of the GM composition with lymphoma and blood cancer, mechanistic understanding of how the microbiota directly or indirectly impacts hematopoiesis is limited.

No studies have evaluated the relationship between GM and therapy outcome in patients with lymphoproliferative disorders treated with chemo-free therapies. Evidence on the relationship between GM and MT in this set of patients is lacking.

Further studies should be considered to open up the possibility for more tailored approaches, in terms of precision medicine, that consider the systemic impact of GM. 

## Figures and Tables

**Table 1:** Described associations between specific microorganisms, and lymphoid malignancies.

Disease	Microorganism	Reference
Gastric MALT	Linked to infection with *Helicobacter pylori*.	[39]
Marginal zone lymphomas	*Hepatitis C virus* (HCV) is a trigger of initial antigenic stimulus for B-cell clonal expansion.	[40]
Burkitt lymphoma	Lymphoma, especially in endemic cases in sub-Saharan Africa, is associated with EBV infection	[41]
Castleman disease	Human herpesvirus 8 (Kaposi sarcoma-associated herpes virus) sequences have been described in some cases of multicentric Castleman disease.	[42]

Modified from Manzo and Bhatt [19].

## Abbreviations

CTLA-4	cytotoxic T-lymphocyte-associated protein 4
C4	Complement component 4
EBV	Epstein-Barr virus
GM	gut microbiota
GPR41-GPR43	G protein-coupled receptors
HCT	hematopoietic cell transplantation
HCV	hepatitis C virus
HHV-8	human herpesvirus 8
HIV	human immunodeficiency virus
HMP	Human Microbiome Project
IL10	Interleukin 10
IFN	Interferon
irAEs	immune related adverse events
LBP	LPS binding protein
LPS	lipopolysaccharide
MALT	mucosal-associated lymphoid tissue
MD	Microbial translocation
MS	multiple sclerosis
NF-kB	nuclear factor kappa-light-chain-enhancer of activated B cells
NGS	next generation sequencing
NOD-1	Nucleotide-binding oligomerization domain-containing protein 1
NSCLC	non–small cell lung cancer
PAMPs	pathogen associated molecular patterns
PD-1	programmed cell death protein 1
PD-L1	programmed cell death protein 1 ligand
PTLD	post-transplantation lymphoproliferative disorder
ROS	reactive oxygen species
sCD14	soluble CD14
SCFAs	short-chain fatty acids
SPF	specific pathogen-free
TLRs	Toll-like receptors
Th1-Th17	T helper cells
Treg	regulatory T cell
VLP	virus-like particles
